# Isolation of alpha-linolenic acid from *Sutherlandia frutescens* and its inhibition of *Mycobacterium tuberculosis’* shikimate kinase enzyme

**DOI:** 10.1186/s12906-016-1344-1

**Published:** 2016-09-17

**Authors:** Peter Masoko, Itumeleng H. Mabusa, Rachmond L. Howard

**Affiliations:** 1 Department of Biochemistry, Microbiology and Biotechnology, University of Limpopo, Private Bag X1106, Sovenga, 0727 South Africa; 2 University of Mpumalanga, C/O R40 White River Rd & D725 Rd, Riverside, Private Bag X11283, Mbombela, 1200 South Africa

**Keywords:** *Sutherlandia frutescens*, *Mycobacterium tuberculosis*, Shikimate kinase

## Abstract

**Background:**

*Sutherlandia frutescens* (L) R.Br. is one of traditional herbal medicines that formed the basis of primary health care systems since the earliest days and is still widely used. *Sutherlandia* is prescribed for people with tuberculosis (TB), but is still not known which compound(s) acts against *M. tuberculosis* and its mode of action. The aim of this study was to identify and isolate antimycobacterial compounds from *S. frutescens* extract*s* against shikimate kinase, a drug target for *M. tuberculosis*.

**Methods:**

*S. frutescens* were dried, ground and extracted with ethanol, dichloromethane: methanol and water. Fractionation and separation of compounds was done with column chromatography. Chromatograms were developed in butanol/acetic acid/water (BAW) [21:6:3]; chloroform/methanol/water/formic acid (CMWF1) [60:15:2:1] and (CMWF2) [21:9:1:0.3]. Separation and isolation of active compounds were done using preparative HPLC. The activity of the plant extracts were also screened against shikimate kinase enzyme (MtbSK) using the MtbSK inhibition assay.

**Results:**

The DCM: MeOH (1:1) extract showed a high percentage inhibition (with an IC_50_ of 0.1 μg/ml) of MtbSK and the purified inhibitor was an Alpha-Linolenic Acid (ALA) compound and it had a significant IC_50_ of 3.7 μg/ml.

**Conclusions:**

This study demonstrated that ALA from *S. frustescens* is an inhibitor of shikimate kinase a good drug target for *M. tuberculosis*.

## Background

*Sutherlandia frutescens* (L) R.Br. is one of traditional herbal medicines that formed the basis of primary health care systems since the earliest days and is still widely used [1]. *S. frutescens* contains several essential, bioactive compounds with clinically proven pharmacological activities [2–4]. This makes the plant attractive as a medicine for various ailments and diseases. *Sutherlandia* is recommended by the South African Department of Health as a supporting treatment for people living with Acquired Immune Deficiency Syndrome (AIDS) as immune system booster [5, 6]. It is also prescribed for treatment of cancer, tuberculosis (TB), diabetes, anxiety and clinical depression [6–8]. TB is a highly infectious disease caused by *Mycobacterium tuberculosis* and it kills millions of people annually. It is also one of the common co-infections in people living with HIV/AIDs thus worsening the HIV/AIDS pandemic.

New drugs with novel mechanisms of action are needed to avoid the cross-resistance problem and more importantly to kill persister TB populations [9]. Advances in molecular tools make it possible for identification of targets essential for survival and persistence whose inhibition is likely to shorten therapy. A study done by Zhang, [10] reviewed various new drug targets and drug candidates; among these was the enzyme shikimate kinase. Shikimate kinase is the fifth enzyme in the shikimate biosynthetic pathway from *M. tuberculosis*. This enzyme is considered an excellent target for developing novel anti-tuberculosis agents as the pathway in which it is involved only takes place in microbes and some plants but is absent in mammals [11]. The shikimate pathway involves seven enzymatic steps in the biosynthesis of chorismate end product, which in turn serves as the precursor for the synthesis of the aromatic amino acids, folates, uniquinones, mycobactins, menaquinones and napthoquinones [12]. The importance of this pathway has been proven in culture using mutants whose growth is completely inhibited without the provision of aromatic supplements [13]. This study was aimed at investigating the activity of *S. frutescens* extracts against *Mycobacterium tuberculosis* shikimate kinase enzyme (MtSK).

At the same time this research seeks to discover new drugs from *S. frutescens* and hence contribute to the fight against TB. There is little scientific data on the activity on *S. frutescens* against TB. The only data available is mainly from personal communications and laboratory reports, on the use of *S. frutescens* plant extract for support treatment in patients suffering from TB. The wide use of *Sutherlandia* in traditional medicine and these preliminary findings suggest that *S. frutescens* is a good candidate for discovery of new anti-mycobacterial drugs which can be used for treatment of TB.

## Methods

### Plant collection

About 1 kg of fresh aerial parts (the leaves and stems) of *Sutherlandia* were collected at a community-based farm in Petrusburg in the Free State, South Africa (29° 6.774′ S; 25° 24.305′ E; 1249 m above sea level). A twig containing a flower was sent to the South African National Biodiversity Institute (SANBI) for identification (SANBI voucher specimen number: 428679). The plant material was finely ground and stored at a room temperature until tests were done.

### Extraction procedure

#### Plant material extraction based on the traditional method

Dried ground aerial parts (leaves and stems) of *S. frutescens* (50 g) were boiled in 2 L of distilled water using a hot plate and a steel extraction vessel covered with an aluminium foil, it was stirred occasionally. The suspension was then removed from the hot plate, cooled in room temperature and filtered through Whatman no.1 filter paper and collected in a glass beaker. The aqueous extraction was freeze-dried and a powder was subsequently obtained. The extract was stored in an airtight container in the cold room at 4 °C until further testing.

#### Plant material extractions using organic solvents

The dried and ground aerial parts of the plant (100 g) were separated into 2 times 50 g each. One litre of 96 % Ethanol was added to 50 g plant material stirred and left overnight. The suspension was filtered the following day using Whatman no.1 filter paper and evaporated to give an ethanol extract (excess ethanol from the Whatman no.1 extract was dried using a fume-hood overnight). The remaining 50 g plant material was used to prepare 1:1 dichloromethane: methanol (DCM:MeOH; 1.4 L) extract. The same procedure used to prepare the ethanol extract was used to prepare the DCM:MeOH; 1:1 extract.

### Shikimate kinase enzyme inhibition assay

A purified shikimate kinase enzyme was obtained from Prof Kenyon at The Council for Scientific and Industrial Research (CSIR) in South Africa and stored at −80 °C was used for the assay and the stock solution was prepared as per the Table 1.

**Table 1:** Stock solution for assay

	Stock solution concentration (mM)	Volume (μl)	Final concentration (mM)
K-PO4 buffer, pH 6.8	250	4760.0	100
MgCl2	60	39.7	0.2
ATP	60	39.7	0.2
Inhibitor	12	991.7	1
KCl	100	1190.0	10
Shikimic acid	100	238.0	2
Enzyme/dH20(nM)	100	1785.0	15
nH2O		2856.0	

**Fig. 1:**
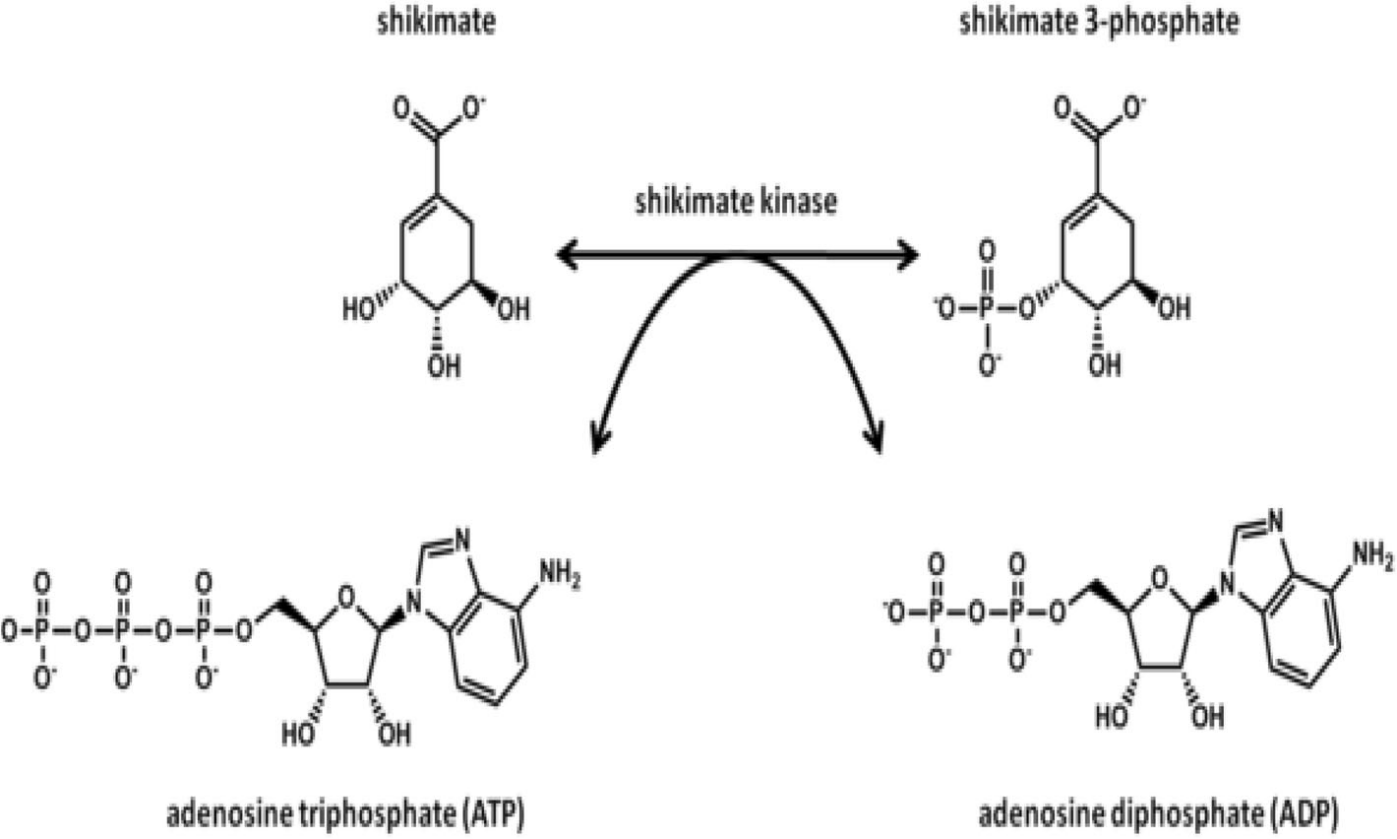
The fifth step of the shikimate pathway (http://en.wikipedia.org/wiki/Shikimate_kinase)

The stock solution containing everything excluding the inhibitor (extracts or fractions) and the ATP was prepared. Extracts/fractions were weighed out to a concentration of 10 mg/ml. Six Eppendorfs were prepared with 1:10 increasing dilution of the extract/fraction. To each Eppendorfs 319 μl of stock solution was added plus the 25 μl of the inhibitor at each concentration. To the blank 25 μl of nH_2_O was added and 1.3 μl of 200 mM EDTA was also added to this tube as a stopping reagent. To the rest of the tubes having reaction mixtures and also to the blank 1.4 μl of 60 mM ATP was added, vortexed and incubated for 15 min. HPLC vials were then lined up, labeled and 319 μl was distributed into each triplicates. The reaction was started by the addition of 1 μM concentration of the enzyme. The assay was done at 37 °C for 15 min. The reaction was terminated by the addition of 2.5 μl 200 mM EDTA. The samples were centrifuged for 2 min at 13,000 rpm and the ADP and ATP concentrations were determined by HPLC. The shikimate kinase inhibition assay is based on quantification of dissociated ADP from the ATP molecule after its phosphate group has been transferred to shikimate as shown on Fig. 1. The IC_50_ for the extracts or fractions were determined by plotting and evaluating the data using Graph Pad prism software, version 5.0. There are no positive controls of known approved shikimate kinase inhibitors used to compare the obtained results with because this study was the first using this approach.

### Fractionation of extracts using column chromatography

The solvent-solvent fractionation was selected to simplify extracts by fractionating the chemical compounds into broad groups based on their solubility. The fractions collected were concentrated using the rotary evaporator (Buchi Rotavapor) under reduced pressure, rotating at 100 rpm and with water bath at 40 °C. The fractions were phytochemically analyzed using TLC. The separated components were visualized with ultraviolet light (360 nm); the plates were sprayed with vanillin-sulphuric acid reagent and heated at 110 °C for colour development.

#### Fractionation of the plant extracts was done as follows:

##### DCM: MeOH 1:1 and ethanol extracts

One gram of each extract was dissolved in 100 % DCM, mixed with silica gel and placed in a warm water bath until it has dried up. A column was packed with 118 g silica gel and 100 % DCM was used to dissolve the silica gel and fractions were eluted as follows:

- Fraction 1 with 100 % DCM yields A
- Fraction 2 with 30 % MeOH/DCM yields B
- Fraction 3 with 70 % MeOH/DCM yields C
- Fraction 4 with 100 % MeOH yields D

##### H_2_O extract

One gram was dissolved in 100 % MeOH and mixed with silica gel and placed in warm water bath until it has dried up. A column was packed with 120 g silica gel dissolved in 100 % MeOH and fractions were eluted as follows:

- Fraction 1 with 30 % DCM/acetone yields E
- Fraction 2 with 100 % ethylacetate yields F
- Fraction 3 and 4 with 100 % MeOH yields both G and H

All collected fractions were left to dry overnight under the fume hood. The fractions with the compound(s) of interest were pooled and preparative HPLC was run for further fractionation and further bioassays.

### Using preparative HPLC to separate the combined fractions A&B

The DCM: MeOH fractions A and B produced in section 2.4 were combined to yield a mass of 2.381 g. A 100 mg of these were dissolved in 5 ml methanol. Two HPLC pumps shown on Tables 2 and 3 below were used. A total of 20 sub-fractions were collected and these 20 sub-fractions were assayed against *M. tuberculosis* shikimate kinase enzyme. The most active sub-fraction was found to be sub-fraction 8.

**Table 2:** Gradient Table for Load pump

Time (min)	Flow (mL/min)	%A IPA	%B MeOH	%C HOH	%D CAN
0.0	1.0	0	100	0	0
25.0	1.0	0	50	0	50
30.0	1.0	0	0	0	100
31.0	1.0	100	0	0	0
38.0	1.0	100	0	0	0
40.0	1.0	0	100	0	0
50.0	1.0	0	100	0	0

**Table 3:** Gradient Table for Gradient Pump

Time (min)	Flow (mL/min)	% HOH	% MeOH	% 0.1 % FA HOH	% CAN
0.0	5.5	0	65	35	0
2.0	5.5	0	65	35	0
15.0	5.5	0	75	12	13
25.0	5.5	0	0	0	100
40.0	5.5	0	0	0	100
44.0	5.5	0	65	35	0
50.0	5.5	0	65	35	0

Column heater temperature: 60 °C

PDA: Scanning 210–600 nm

Collection: Hold time: 1 min, 2 min collection intervals for 20 vials

## Results and discussion

**Fig. 2:**
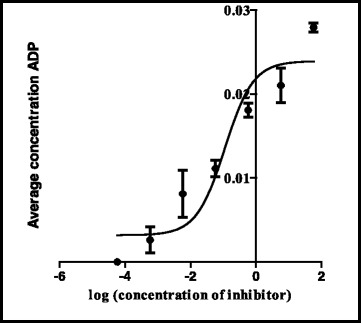
Effect of the DCM: MeOH 1:1 extract on the IC_50_

**Fig. 3:**
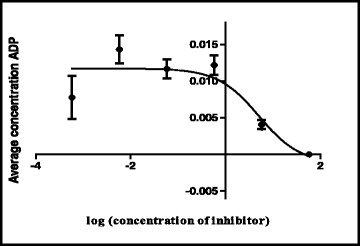
Effect of the H_2_O extract on the IC_50_

**Fig. 4:**
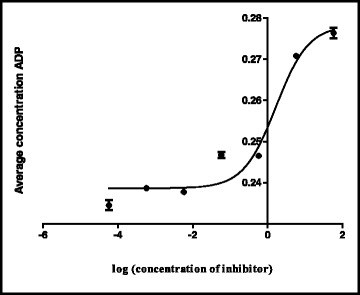
Effect of the EtOH extract on the IC_50_

The shikimate enzyme is one of the most vital enzymes involved in the metabolic pathways of *Mycobacterium tuberculosis*. The principle of the shikimate kinase inhibition assay is primarily based on the enzyme shikimate kinase phosphorylating shikimic acid to form shikimate-3-phosphate and a resultant ADP as shown in Fig. 1. In the presence of a good inhibitor less or none of this resultant ADP will be produced, an appropriate inhibitor would bind where a phosphate is supposed to bind on the shikimic acid and therefore subsequently restricting its phosphorylation and hence producing no ADP. The strongest inhibitor produces less ADP. After performing a series of shikimate kinase inhibition assays on the crude extracts it was noticed that the DCM: MeOH 1:1 extract was showing more potential in terms of inhibition of the shikimate kinase enzyme’s activity and hence had a fairly low average of ADP produced and also as seen on Table 4 had an IC_50_ of 0.1 ug/ml (Fig. 2) which was lower than the H_2_O (5.1 ug/ml) (Fig. 3) and the ethanol (1.7 ug/ml) extracts (Fig. 4). This realization led to the decision to performing more tests with this extract.

**Table 4:** IC_50_ values of all the extracts, fractions and sub fractions

Sample name	IC_50_ value (μg/ml)
*S. frutescens* crude extracts (January)	
H_2_O	5.1
EtOH	1.7
DCM: MeOH 1:1	0.1^a^
DCM: MeOH 1:1 fractions	
fraction A	2.5^a^
fraction B	0.3^a^
fraction C	1.5
fraction D	94.3

^a^The extract or fraction was chosen for further analysis

**Fig. 5:**
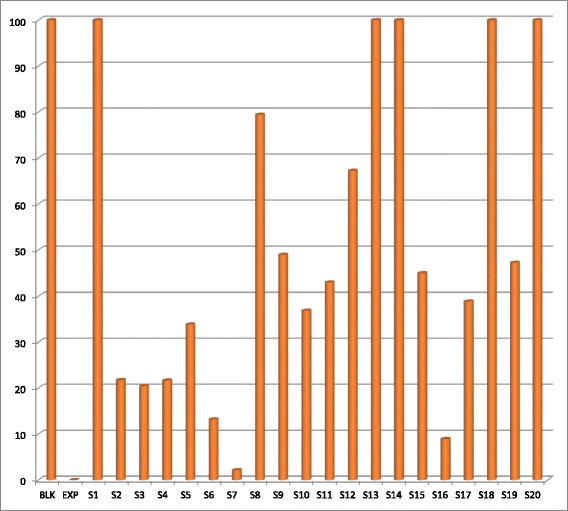
Chart representing percentage inhibition of the single point inhibitions of the 20 sub-fractions produced via preparatory HPLC

**Fig. 6:**
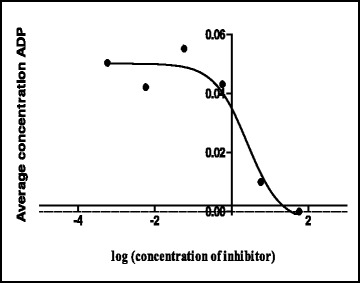
Dose response curve determining the IC_50_ of sub-fraction 8 from the fractionation of fractions A&B of the June DCM: MeOH 1:1 extract

**Fig. 7:**
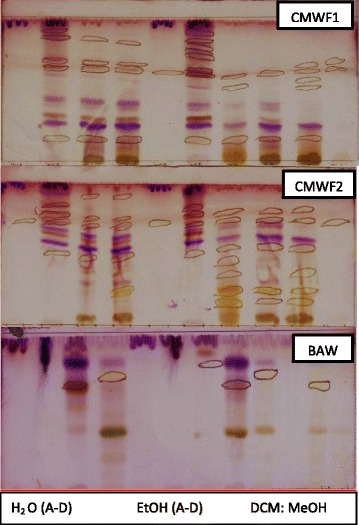
TLC plates developed in different mobile phases. The TLC plates were sprayed with 0.1 % vanillin-sulphuric acid. From left to right, the first four lanes represent the H_2_O fractions, the next four lanes represent EtOH fractions and the last four lanes represent DCM:MeOH (1:1) fractions

Percentage inhibition obtained from single point inhibitions of the 20 sub-fractions produced via preparative HPLC. Sample 1,13,14,18 and 20 shows almost 100 % inhibition while sample 7 has almost 0 % inhibition against shikimate kinase (Fig. 5). Of the 20 sub-fractions that were collected, sub-fraction 8 was shown to be one of the most active against the shikimate kinase enzyme (Fig. 6), hence the decision to pursue its isolation and characterization. Figure 6 shows that at just after 10 min, a large peak of a compound (sub-fraction 8) with a molecular ion peak at (*m/z)* 277.4 was collected and this compound ionized in the negative electrospray ionization (ES-). This compound had a bright yellow colour and was sticky upon evaporation with the rotavap. The TLC plate on Fig. 7 also showed a single band, meaning that separation did occur and that a single compound was found in this sub-fraction 8. The most useful tool in identification of a compound in chemistry is the SDBS assimilations software; this software was very helpful in determining the structure and molecular formula of the isolated compound by using its proton and carbon NMR spectra (Table 5). The protons and the carbons of the isolated compound exactly matched those assimilated with from the software hence the software was able to identify the compound’s chemical formula as C_18_H_30_O_2_ giving a total molecular mass of 278 *u*.

**Table 5:** Spectroscopic data of the SDBS ALA assimilations and the NMR from sub-fraction 8

^13^C	SDBS	Sub-fraction 8
1	180.55	
2	131.94	131.97
3	130.23	130.25
4	128.30	128.30
5	128.30	128.26
6	127.84	127.76
7	127.19	127.13
8	34.17	34.01
9	29.63	29.56
10	29.19	29.14
11	29.13	29.07
12	29.13	29.04
13	27.26	27.17
14	25.68	25.62
15	25.60	25.54
16	24.72	24.72
17	20.61	20.54
18	14.30	14.24

**Fig. 8:**
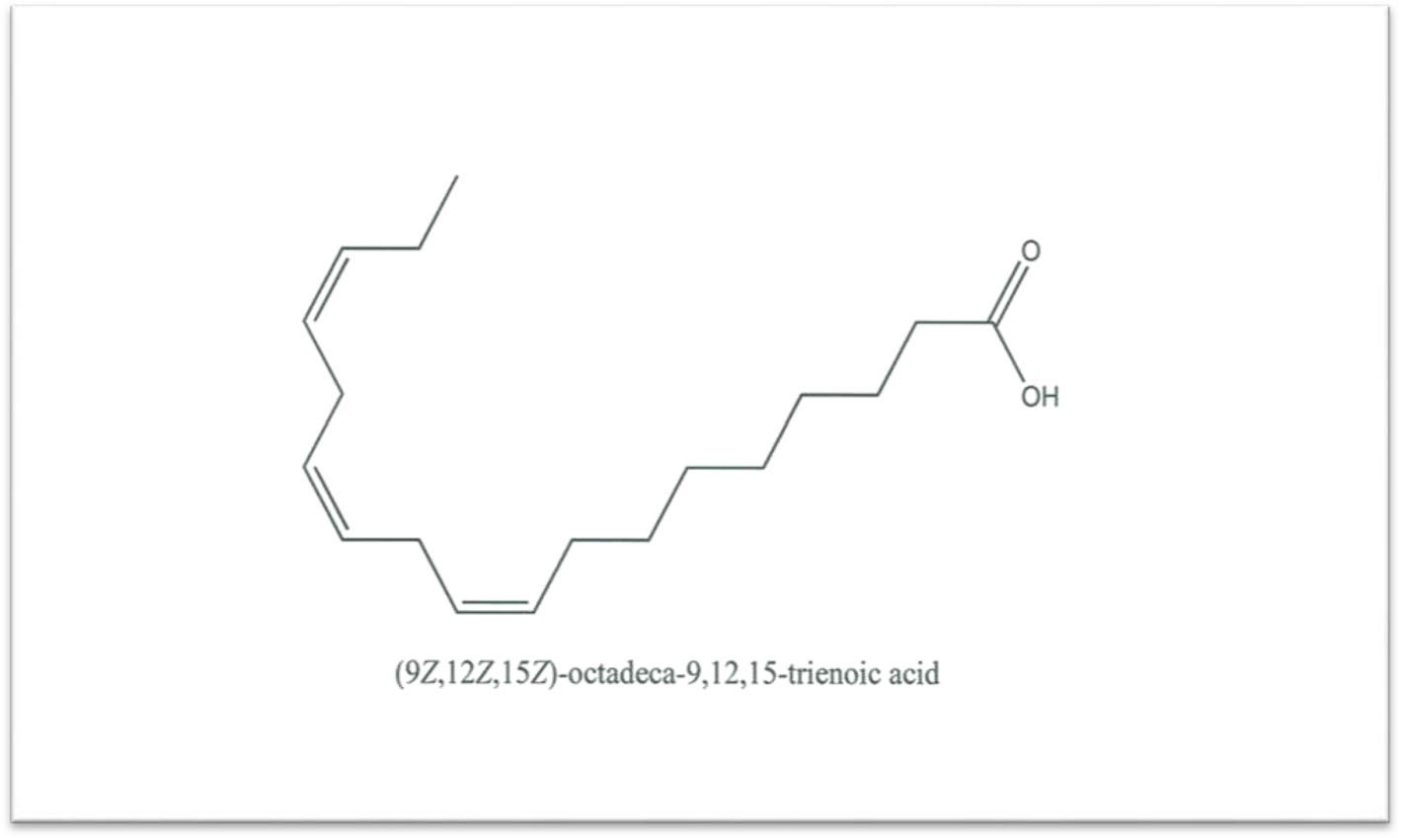
Structure of the isolated compound (sub-fraction 8) and its IUPAC name

**Fig. 9:**
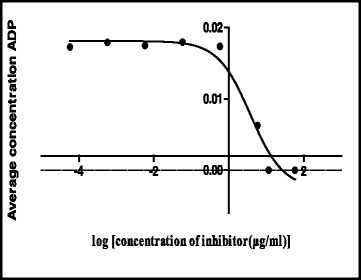
IC_50_ data of ALA from the DCM: MeOH 1:1 extract’s fractions

Its IUPAC name and structure were determined using Chem Draw ultra 8 software and these are represented on Fig. 8. The IUPAC name of the isolated compound was found to be (9*Z*, 12*Z*, 15*Z*)-octadeca-9,12,15-trienoic acid and its common name is alpha linolenic acid. IC_50_ of the compound was also determined and recorded to be 3.7 ug/ml (Fig. 9). Alpha-linolenic (ALA) is an essential omega-3 fatty acid that cannot be synthesized by the human body and hence has to be supplied by dietary sources such as fish, plants, walnuts, grape seed (canola), several legumes, flaxseed, and green leafy vegetables and vegetable oils [14, 15] but has not been reported to have been isolated from *S. frutescens* before. Stark et al. [14] mentioned that potential benefits of ALA include cardio-protective effects, modulation of the inflammatory response, and a positive impact on both central nervous system function and behavior. According to Stark et al. [14], as a result of many studies they have conducted in their study, ALA is not only essential for dietary requirements but also has therapeutic properties. Several studies have proven amongst other free fatty acids that alpha linolenic acid is active against a wide range of microorganisms including bacteria such as *S. aureus* and *Bacillus subtilis* [16], *Helicobacter pylori*, [17] viruses such as hepatitis C virus (HCV) [18] and fungi such as *Rhizoctonia solani* and *Crinipellis perniciosa* [19]. According to Desbois and Smith, [20] their antibacterial mode of action is not clearly understood but may also result from the inhibition of enzyme activity, impairment of nutrient uptake, generation of peroxidation and auto-oxidation degradation products or direct lysis of bacterial cells. The broad spectrum of activity they possess, the non-specific mode of action and their safety makes them attractive as antibacterial agents for various applications in medicine, agriculture and food preservation.

This is a novel study. Firstly, this is the first report on identifying ALA from *S. frutescens.* Secondly, this is the first report showing ALA from *S. frutescens* inhibiting shikimate kinase enzyme, an important drug target for *M. tuberculosis*. Thirdly, this is the first time that ADP quantification method for shikimate kinase inhibition assay using HPLC was used. In other words, a reliable HPLC based *M. tuberculosis* shikimate kinase inhibition assay was developed in this study. Several studies have used liquid chromatography-Mass spectrometry (LC-MS) to determine *M. tuberculosis* shikimate kinase enzyme activity by quantifying shikimate-3-phosphate which is a resulting product of the phosphorylation of shikimate [21–23]. The advantage of using the ADP method is that one can see the viability of the enzyme by tracing the production of ADP thus using the HPLC machine not only ensures the collection of qualitative data but also the quantitative data. The ADP method used in this study could be useful for scientists to study other potential *M. tuberculosis* shikimate kinase inhibitors. Kinases have a conserved phosphoryl transfer mechanism [24–26] thus to find a unique inhibitor and a way of determining inhibition of kinases advance kinases kinetic studies.

## Conclusion

In conclusion, this study demonstrated that ALA from *S. frustescens* is an inhibitor of shikimate kinase a good drug target for *Mycobacterium tuberculosis*. Further studies to test the isolated compound, ALA on whole *M. tuberculosis* cells will be conducted.

## Abbreviations

AIDS: Acquired immune deficiency syndrome
ALA: Alpha-linolenic acid
BAW: Butanol/acetic acid/water
CMWF: Chloroform/methanol/water/formic acid
HPLC: High performance liquid chromatography
IUPAC: International Union of pure and applied chemistry
MtbSK: Shikimate kinase enzyme
TLC: Thin layer chromatography
